# Assessing the impact of transfusion thresholds in patients with septic acute kidney injury: a retrospective study

**DOI:** 10.3389/fmed.2023.1308275

**Published:** 2023-12-21

**Authors:** Xiangyuan Ruan, Baoxin Wang, Yifan Gao, Jinmei Wu, Xueshu Yu, Chenglong Liang, Jingye Pan

**Affiliations:** ^1^Department of Intensive Care Unit, The First Affiliated Hospital of Wenzhou Medical University, Wenzhou, China; ^2^Key Laboratory of Intelligent Treatment and Life Support for Critical Diseases of Zhejiang Province, Wenzhou, China; ^3^Wenzhou Key Laboratory of Critical Care and Artificial Intelligence, Wenzhou, China; ^4^Zhejiang Engineering Research Center for Hospital Emergency and Process Digitization, Wenzhou, China

**Keywords:** transfusion threshold, septic acute kidney injury, hemoglobin concentration, S-AKI, sepsis transfusion threshold, sepsis

## Abstract

**Background:**

Sepsis is a severe condition that often leads to complications such as acute kidney injury, which significantly increases morbidity and mortality rates. Septic AKI (S-AKI) is common in ICU patients and is associated with poor outcomes. However, there is no consensus on the optimal transfusion threshold for achieving the best clinical results. This retrospective study aims to investigate the relationship between different transfusion thresholds during hospitalization and the prognosis of septic AKI.

**Methods:**

Data from patients with S-AKI was extracted from MIMIC-IV. Based on the lowest hemoglobin level 24 h before transfusion, patients were divided into high-threshold (≥7 g/L) and low-threshold (<7 g/L) groups. We compared the outcomes between these two groups, including hospital and ICU mortality rates as primary outcomes, and 30 days, 60 days, and 90 days mortality rates, as well as duration of stay in ICU and hospital as secondary outcomes.

**Results:**

A total of 5,654 patients were included in our study. Baseline characteristics differed significantly between the two groups, with patients in the low-threshold group generally being younger and having higher SOFA scores. After performing propensity score matching, no significant differences in survival rates were found between the groups. However, patients in the low-threshold group had a longer overall hospital stay.

**Conclusion:**

A lower transfusion threshold does not impact the mortality rate in S-AKI patients, but it may lead to a longer hospital stay.

## Introduction

Sepsis is a life-threatening condition characterized by a dysregulated immune response to infection (1). Acute kidney injury (AKI) is a severe complication of sepsis, significantly contributes to morbidity and mortality. AKI occurs in approximately 30% to 50% of ICU patients with sepsis (2, 3). AKI presence in sepsis patients can increase in-hospital mortality risk by 6 to 8 times and extend hospital stays (3–5).

Sepsis-associated AKI, or septic AKI (S-AKI), represents a significant global public health challenge (2, 4). Characterized by rapid renal function decline, it leads to metabolic waste accumulation and electrolyte imbalances (4). The pathophysiology of S-AKI is complex and multifactorial, involving inflammation, microvascular dysfunction, and direct tubular injury (2–8). Particularly, hypoxia plays a crucial role in septic AKI’s pathogenesis (9).

Managing S-AKI requires a multidisciplinary approach, including prompt identification and treatment of the underlying infection, hemodynamic optimization, fluid resuscitation, and supportive care (2, 3, 6). du Cheyron et al. (10) reported an association between low initial hemoglobin levels and increased mortality risk in ARF patients. Experimental studies in animals demonstrate that fluid resuscitation (FR) improves hemodynamics but does not ameliorate renal microcirculation dysfunction (11). Blood transfusion, however, can restore renal microcirculation oxygenation in endotoxemia rat models, suggesting its potential benefits in renal function restoration (12).

The influence of transfusion thresholds on S-AKI prognosis remains under-explored. Presently, conclusive evidence is lacking on the optimal transfusion threshold for these patients. Studies indicate similar mortality rates and ischemic event incidences in septic patients with high and low transfusion thresholds (13–18). Consequently, the 2016 and 2021 Surviving Sepsis Campaign guidelines have eliminated higher hemoglobin targets for sepsis resuscitation, recommending a transfusion threshold below 7.0 g/dL (1, 19). There is no universally agreed-upon transfusion threshold specifically tailored for AKI patients (6). Transfusion decisions in AKI patients are multifactorial, depending on the patient’s hemodynamic stability, AKI cause, concurrent conditions, and overall clinical profile. Numerous guidelines support a restrictive transfusion strategy for critically ill patients, AKI patients included (15, 16, 20, 21). Research on the optimal red blood cell transfusion threshold in cardiac surgery-acute kidney injury (CS-AKI) patients suggests that a restrictive strategy (Hb <7.5 g/dL) is comparable to a liberal approach (22).

In S-AKI patients, blood transfusion is commonly used to treat anemia, aiming to enhance tissue oxygenation and offset anemia. However, the optimal transfusion threshold for these patients is still undefined. Although various studies suggest different transfusion thresholds, a consensus on the optimal level for the best clinical outcomes is yet to be established.

Consequently, this retrospective study aims to evaluate the relationship between transfusion thresholds (≥7 g/dL and <7 g/dL) and S-AKI prognosis. Our goal, through analyzing a large cohort of S-AKI patients, is to ascertain the link between higher or lower transfusion thresholds and improved clinical outcomes. This study aims to bridge the existing knowledge gap and offer significant insights into S-AKI management.

## Methods

### Database

This investigation adheres to the guidelines outlined in the strengthening the reporting of observational studies in epidemiology (STROBE) statement (23). Data was extracted from the publicly available Medical Information Mart for Intensive Care (MIMIC)-IV database, which contains comprehensive hospitalization records. This relational database includes genuine hospital stays for patients admitted to a tertiary academic medical center (Beth Israel Deaconess Medical Center) in the United States. It covers 76,540 ICU admissions related to 53,150 patients spanning the period from 2008 to 2019.

### Ethical considerations

This research adhered to the ethical principles outlined in the Declaration of Helsinki and received approval from the Institutional Review Boards of both MIT and Beth Israel. The author XR completed the necessary training for using this database and obtained certification (Record ID 42912881).

### Study cohort

We included all patients with suspected sepsis who met the diagnostic criteria for infection, as indicated by a sequential organ failure assessment (SOFA) score ≥2, based on the third international consensus definition of sepsis and septic shock (Sepsis-3.0) (24). The diagnosis of AKI was based on elevated serum creatinine levels and/or reduced urine output, following the KDIGO (kidney disease improving global outcomes) guidelines. AKI staging was determined using the KDIGO serum creatinine criteria (25). We included data from adult patients (aged ≥18 years) who were admitted to the ICU for at least 1 day during their initial hospitalization. Patients who did not receive transfusions were excluded. Subsequently, patients were divided into two groups based on their lowest hemoglobin value 24 h before transfusion: the higher threshold group (hemoglobin level ≥7 g/dL) and the lower threshold group (hemoglobin level <7 g/dL).

### Covariates

Data was extracted from the database using Structured Query Language (SQL). We collected the following demographic parameters: gender, age, race (White, Black, other), SOFA, Charlson comorbidity index myocardial infarct, congestive heart failure, cerebrovascular disease, chronic pulmonary disease, diabetes mellitus, liver disease, renal disease, malignant cancer, vital signs (heart rate, mean arterial pressure, respiratory rate, SpO_2_) and laboratory values (Hb, PLT, WBC, INR, PT, APTT, creatinine, potassium, sodium, bicarbonate, lactate, PO_2_, PCO_2_, pH).

### Outcomes

The primary outcomes of the study were hospital mortality and ICU mortality. The secondary outcomes included 30 days, 60 days, and 90 days mortality, as well as the duration of stay in the ICU and the hospital.

### Statistical methods

Baseline characteristics and measurements of interest that are normally distributed are presented as mean ± standard deviation. Conversely, attributes not adhering to a normal distribution are represented by the median (M) and interquartile range (IQR). For comparative purposes, chi-square tests, one-way ANOVA, and Kruskal–Wallis tests were utilized to compare categorical, normally distributed, and non-normally distributed continuous variables, respectively. A multivariate Cox regression was employed to discern the correlation between two patient cohorts and the primary outcome.

To mitigate potential bias arising from missing data, the main outcome analysis was carried out after imputation of data using the Random Forest methodology. Propensity score matching (PSM), known for its ability to adjust for intergroup variations and eliminate bias in non-randomized comparative studies (26), was extensively implemented. In this study, a 1:1 matching was achieved through the optimal matching algorithm. Both Cox regression and PSM were utilized to adjust covariates, based on a nearest neighbour algorithm and a caliper width of 0.2 (27); all variables were adjusted via PSM. The standardized mean difference (SMD) was computed to assess the effectiveness of the PSM, with a threshold less than 0.1 considered acceptable. Within the matched cohorts, both the Kaplan–Meier method and the Cox proportional hazards regression were employed to compare hospital mortality, ICU mortality, and 30 days, 60 days, and 90 days mortality. The Wilcoxon rank-sum test was used to determine whether there was a significant difference in the median duration of stay in the ICU and the hospital between the two groups. Statistical significance was set at a two-sided *p*-value <0.05.

To mitigate the possibility of bias from the designated treatment and confounding conditions, all statistical analyses in our research was undertaken rendering to the provisions of R software, version 4.2.2.

## Results

### Basic information

**Figure 1:**
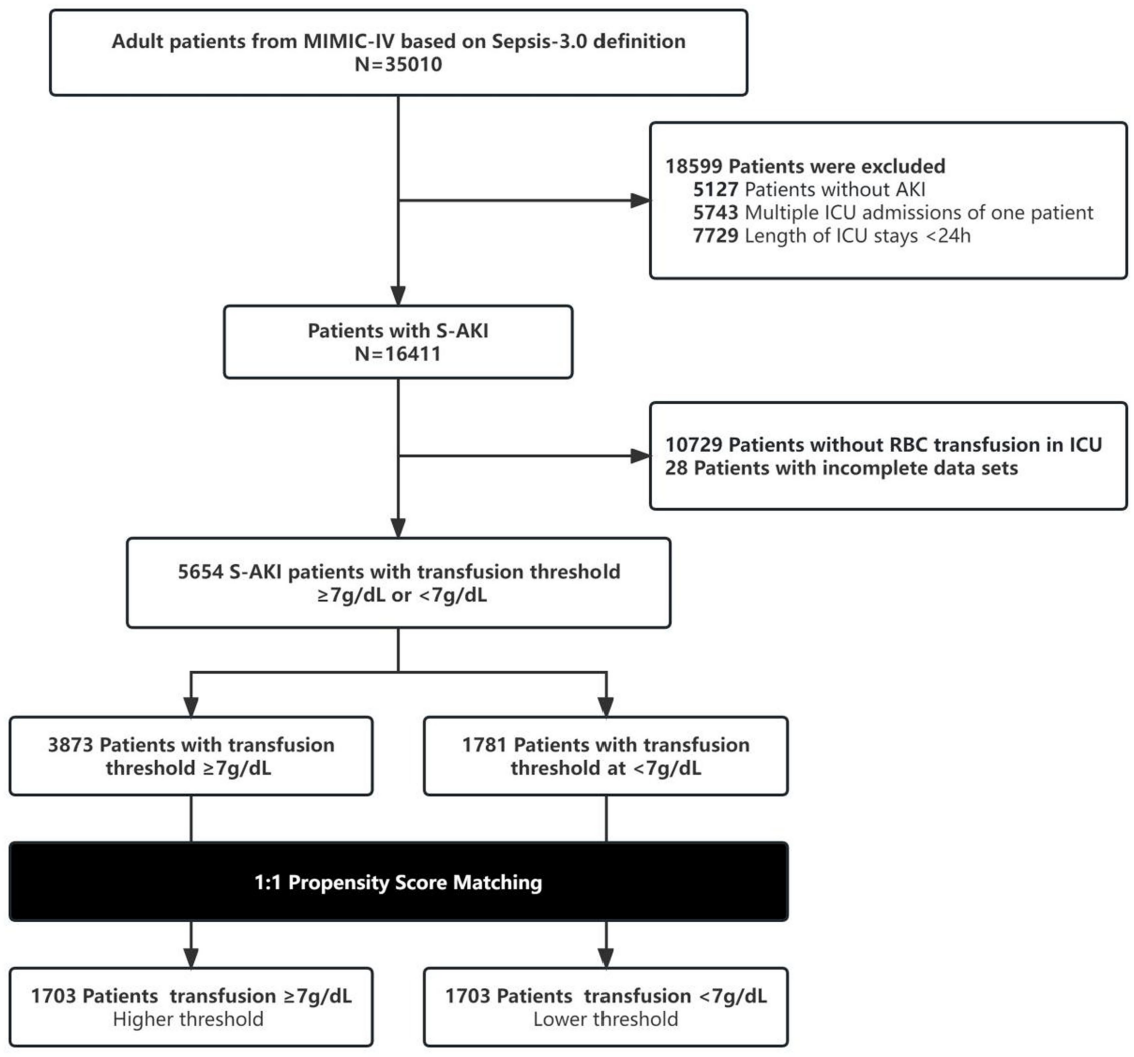
Flow diagram for data inclusion in the present study.

After applying the exclusion criteria, a total of 5,654 patients were included in the original cohort, with 3,873 in the high threshold group and 1,781 in the lower threshold group. After performing PSM, successful matches were obtained for both the higher and lower threshold groups, resulting in 3,406 patients included in the current investigation. An flowchart depicting the screening and matching process is presented in the designated Figure 1. Demographic characteristics (such as gender, age, and ethnicity), SOFA scores, vital signs, concurrent medical conditions, and laboratory test results are compared before and after matching, as shown in Table 1. In the original cohort, patients in the low threshold group were relatively younger and had higher SOFA scores, as well as a higher incidence of comorbidities, compared to the high threshold group. A significant difference in certain baseline features was observed between the two groups prior to PSM. Using PSM in a 1:1 ratio, 1,703 patients in the low threshold group were matched with an equal number of patients in the high threshold group (Table 1). A graphical representation demonstrating the comparison curves of weighted data pre and post-matching is illustrated in Supplementary Figure S1.

**Table 1:** Baseline characteristics of the patients.

Variables	Original cohort				Match cohort			
	Higher threshold	Lower threshold	*p*-value	SMD	Higher threshold	Lower threshold	*p*-value	SMD
*n*	3,873	1,781			1,703	1,703		
Age (years)	67.47 ± 15.17	65.01 ± 16.23	<0.001	0.152	65.23 ± 15.89	65.07 ± 16.26	0.775	0.010
Male, *n* (%)	2,236 (57.7)	882 (49.5)	<0.001	0.164	853 (50.1)	852 (50.0)	1.000	0.001
Ethnicity (%)			<0.001				0.966	
White	2,680 (69.2)	1,108 (62.2)		0.144	1,078 (63.3)	1,072 (62.9)		0.007
Black	238 (6.1)	186 (10.4)		0.141	164 (9.6)	168 (9.9)		0.008
Other	955 (24.7)	487 (27.3)		0.060	461 (27.1)	463 (27.2)		0.003
AKI stage (%)			<0.001				0.978	
1	681 (17.6)	286 (16.1)		0.042	270 (15.9)	274 (16.1)		0.006
2	1,725 (44.5)	627 (35.2)		0.195	609 (35.8)	610 (35.8)		0.001
3	1,467 (37.9)	868 (48.7)		0.217	824 (48.4)	819 (48.1)		0.006
SOFA	4.18 ± 2.39	4.55 ± 2.65	0.001	0.138	4.47 ± 2.63	4.51 ± 2.62	0.647	0.015
*Vital signs*								
Heart rate (bpm)	88.72 ± 16.58	91.25 ± 16.46	<0.001	0.154	91.26 ± 17.74	91.25 ± 16.45	0.984	0.001
Respiratory rate (bpm)	18.94 ± 4.59	20.34 ± 4.65	<0.001	0.302	20.22 ± 4.85	20.31 ± 4.65	0.600	0.018
MAP (mmHg)	74.70 ± 11.59	73.64 ± 10.70	<0.001	0.100	73.90 ± 10.95	73.86 ± 10.58	0.931	0.003
SPO_2_ (%)	97.60 ± 2.58	97.36 ± 2.67	<0.001	0.090	97.38 ± 2.23	97.35 ± 2.70	0.672	0.013
*Comorbidities, n (%)*								
Myocardial infarct	847 (21.9)	297 (16.7)	<0.001	0.139	270 (15.9)	285 (16.7)	0.516	0.024
Congestive heart failure	1,192 (30.8)	549 (30.8)	0.996	0.001	501 (29.4)	525 (30.8)	0.390	0.031
Cerebrovascular disease	497 (12.8)	263 (14.8)	0.053	0.055	246 (14.4)	253 (14.9)	0.771	0.012
Chronic pulmonary disease	1,032 (26.6)	457 (25.7)	0.453	0.023	442 (26.0)	441 (25.9)	1.000	0.001
Liver disease	822 (21.2)	462 (25.9)	<0.001	0.108	434 (25.5)	434 (25.5)	1.000	0.000
Diabetes mellitus	1,144 (29.5)	546 (30.7)	0.411	0.024	519 (30.5)	523 (30.7)	0.911	0.005
Renal disease	888 (22.9)	505 (28.4)	<0.001	0.120	458 (26.9)	472 (27.7)	0.617	0.018
Malignant cancer	478 (12.3)	331 (18.6)	<0.001	0.160	306 (18.0)	304 (17.9)	0.964	0.003
*Laboratory tests*								
WBC (10^9^/L)	13.14 ± 8.43	14.12 ± 16.65	0.019	0.059	13.82 ± 10.37	14.06 ± 16.70	0.628	0.014
Platelet (10^9^/L)	179.90 ± 111.96	186.32 ± 141.30	0.091	0.045	188.80 ± 131.08	186.61 ± 140.39	0.639	0.015
INR	1.58 ± 0.72	1.80 ± 1.04	<0.001	0.211	1.73 ± 0.95	1.75 ± 0.92	0.398	0.026
PT (seconds)	17.27 ± 7.05	19.53 ± 10.86	<0.001	0.208	18.68 ± 9.11	19.04 ± 9.76	0.261	0.034
APTT (seconds)	42.98 ± 20.16	44.36 ± 21.68	0.023	0.064	44.13 ± 21.78	43.91 ± 21.02	0.770	0.010
Bicarbonate (mmol/L)	22.69 ± 4.37	21.77 ± 5.41	<0.001	0.170	22.02 ± 4.77	21.88 ± 5.38	0.425	0.026
Potassium (mmol/L)	4.25 ± 0.61	4.29 ± 0.75	0.061	0.051	4.27 ± 0.67	4.29 ± 0.75	0.546	0.020
Sodium (mmol/L)	138.26 ± 4.88	138.52 ± 6.11	0.128	0.041	138.47 ± 5.41	138.43 ± 6.00	0.835	0.007
Creatinine (mg/dL)	1.59 ± 1.48	2.03 ± 2.02	<0.001	0.218	1.93 ± 1.80	1.98 ± 1.97	0.450	0.024
pH	7.37 ± 0.08	7.36 ± 0.09	0.005	0.078	7.37 ± 0.08	7.36 ± 0.09	0.339	0.032
PO_2_ (mmHg)	173.80 ± 87.06	125.56 ± 76.00	<0.001	0.635	132.90 ± 70.20	127.88 ± 76.54	0.046	0.066
PCO_2_ (mmHg)	40.45 ± 6.95	40.15 ± 8.52	0.197	0.035	40.17 ± 8.11	40.24 ± 8.48	0.820	0.008
Lactate (mmol/L)	2.65 ± 1.94	2.85 ± 2.55	0.004	0.077	2.79 ± 2.30	2.81 ± 2.52	0.832	0.007

SMD, standardized mean difference; SOFA, sequential organ failure assessment; MAP, mean arterial pressure; WBC, white blood cell; INR, international normalized ratio; PT, prothrombin time; APTT, activated partial thromboplastin time.

### Hemoglobin concentrations and transfusion

**Figure 2:**
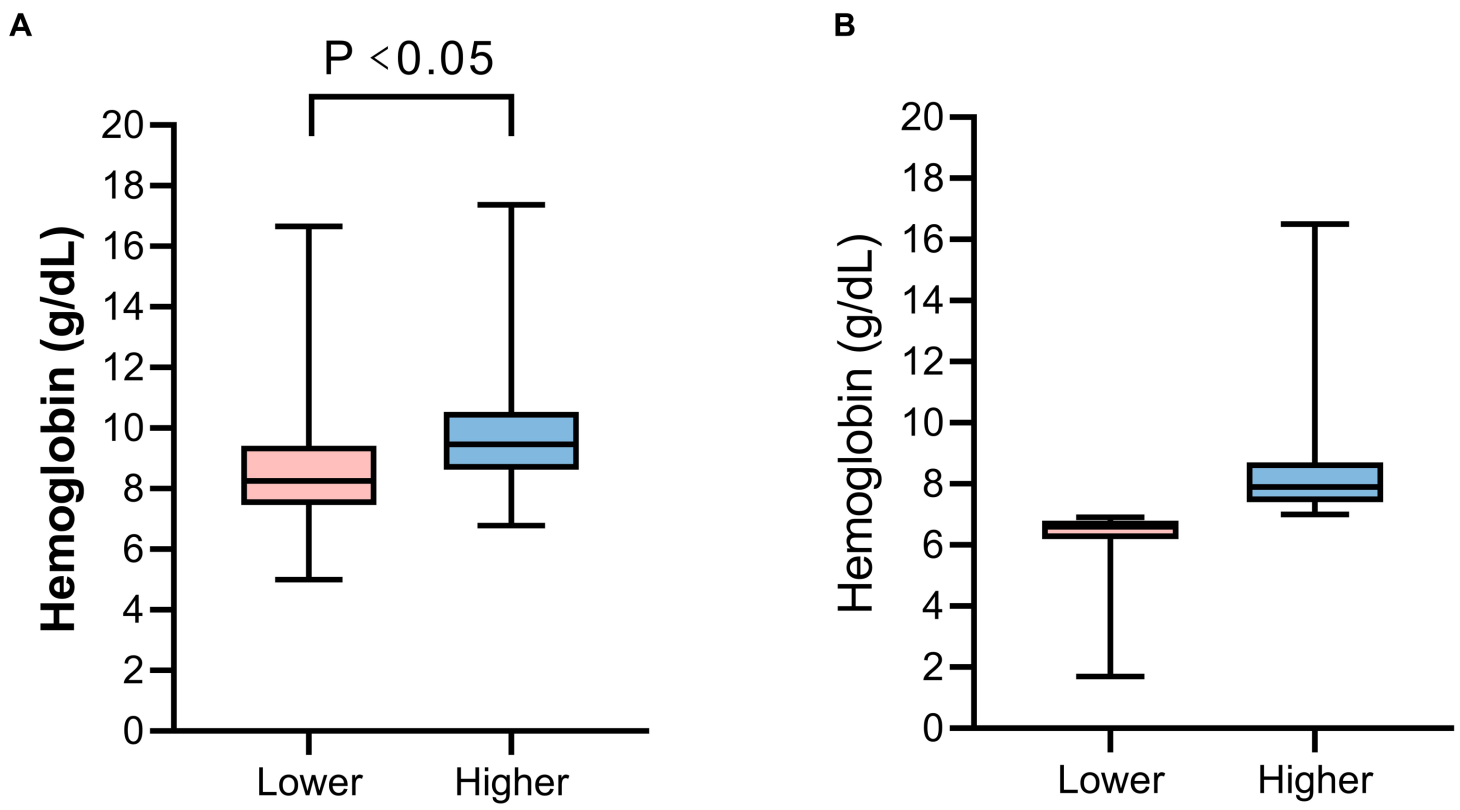
The hemoglobin concentration of patients. **(A)** The hemoglobin concentration upon ICU admission. **(B)** The hemoglobin threshold before blood transfusion between the two groups.

**Figure 3:**
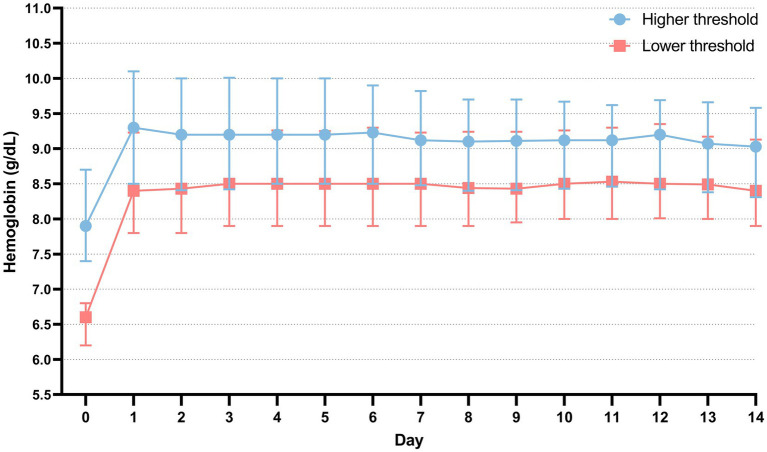
The comparison of hemoglobin concentration trends within 14 days post-transfusion and at ICU admission. Day 0 is the day of ICU admission. Day 1 is defined as the first day after blood transfusion. The data is presented as the median, 25th percentile, and 75th percentile.

The distribution of hospital mortality among patients, stratified by transfusion thresholds, is depicted in Supplementary Figure S2. The hemoglobin threshold for the high-threshold group was 7.9 g/dL [interquartile range (IQR), 7.4–8.7], while the low-threshold group had a threshold of 6.6 g/dL (IQR, 6.2–6.8) (Figure 2B). We also compared the hemoglobin concentrations of patients within the first 24 h after ICU admission. The average hemoglobin concentration upon ICU admission differed significantly between the high-threshold and low-threshold groups [9.8 g/dL (IQR, 8.6–10.5) vs. 8.3 g/dL (IQR, 7.5–9.4), *p* < 0.001] (Figure 2A). The comparison of hemoglobin concentration trends from ICU admission to 14 days post-transfusion is shown in Figure 3. Compared to baseline levels, the hemoglobin concentration significantly increased on the first day post-transfusion (*p* < 0.001), and gradually stabilized in the following days.

### Primary outcome

**Figure 4:**
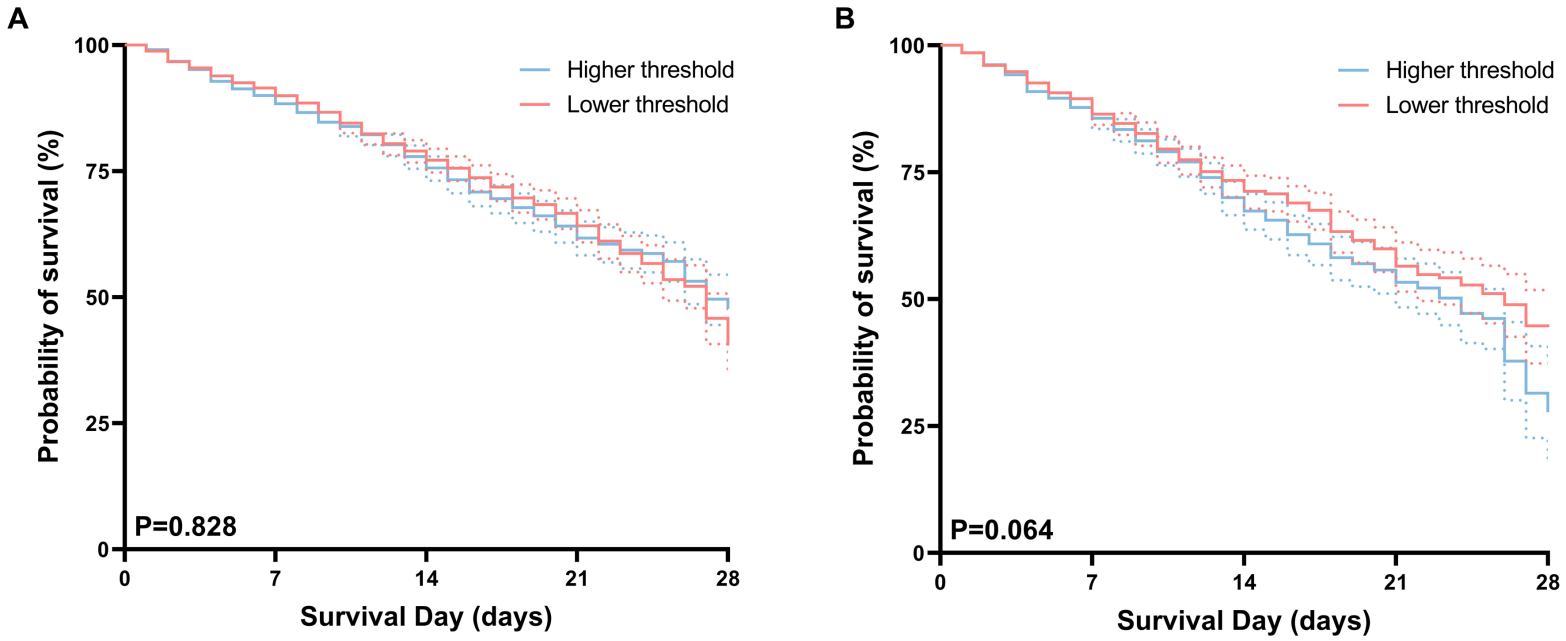
Kaplan–Meier curves for hospital **(A)**, ICU **(B)** survival probability comparing patients with permissive lower threshold and higher threshold. For each curve, 95% confidence intervals (dotted lines) are shown.

After performing PSM, the hospital mortality rates were determined to be 26.1% (445/1,703) in the higher threshold cohort and 27.1% (461/1,703) in the lower threshold cohort. The corresponding ICU mortality rates were determined to be 21.1% (358/1,703) in the higher threshold cohort and 19.3% (328/1,703) in the lower threshold cohort (Table 2). The Kaplan–Meier survival analysis shows no significant differences in survival probabilities between the high-threshold group and the low-threshold group in terms of both hospital mortality and ICU mortality, as illustrated in Figure 4.

**Table 2:** The primary outcomes and secondary outcomes.

Outcomes	Higher threshold	Lower threshold	HR or mean difference (95% CI)	*p*-value
	*n* = 1,703	*n* = 1,703		
*Primary outcome*				
Hospital-mortality	445 (26.1)	461 (27.1)	1.01 (0.89, 1.16)	0.828
ICU-mortality	358 (21.1)	328 (19.3)	1.15 (0.99, 1.34)	0.064
*Secondary outcome*				
30 days mortality	400 (23.5)	410 (24.1)	0.99 (0.86, 1.13)	0.840
60 days mortality	447 (26.2)	468 (27.5)	0.96 (0.85, 1.10)	0.563
90 days mortality	466 (27.4)	483 (28.4)	0.96 (0.86, 1.11)	0.687
ICU length of stay (days)	6.3 (3.2 to 12.9)	6.5 (3.2 to 13.4)	−0.69 (−1.37, −0.01)	0.347
Hospital length of stay (days)	13.8 (8.0 to 23.9)	15.7 (8.7 to 26.9)	−2.29 (−3.59, −1.00)	<0.001

### Secondary outcomes

**Figure 5:**
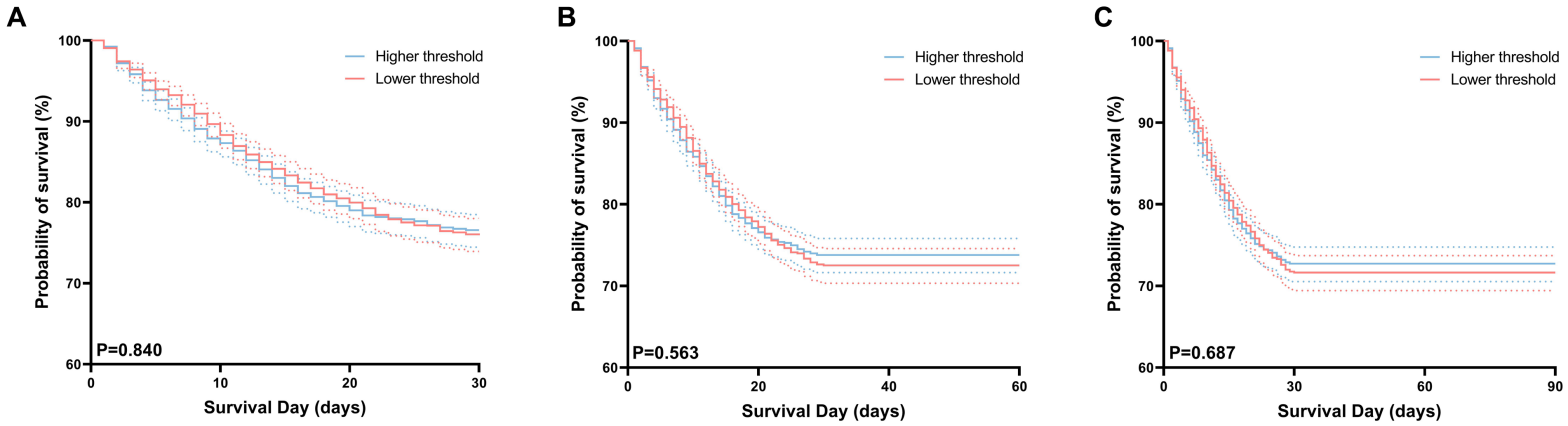
Kaplan–Meier curves for 30 days **(A)**, 60 days **(B)**, 90 days **(C)** survival probability comparing patients with permissive lower threshold and higher threshold. For each curve, 95% confidence intervals (dotted lines) are shown.

There were no substantial differences in 30 days, 60 days, and 90 days mortality between the high-threshold group and the low-threshold group (Figure 5). In this study, we compared the duration of hospital and intensive care unit (ICU) stays between two groups of patients. The results indicate that although there was no significant difference in the duration of ICU stays between the two groups (6.5 vs. 6.3, *p* = 0.347), but patients in the low-threshold group had a significantly longer overall hospital stay compared to their high-threshold group counterparts (15.7 vs. 13.8, *p* < 0.001) (Table 2).

### Subgroup analysis

**Figure 6:**
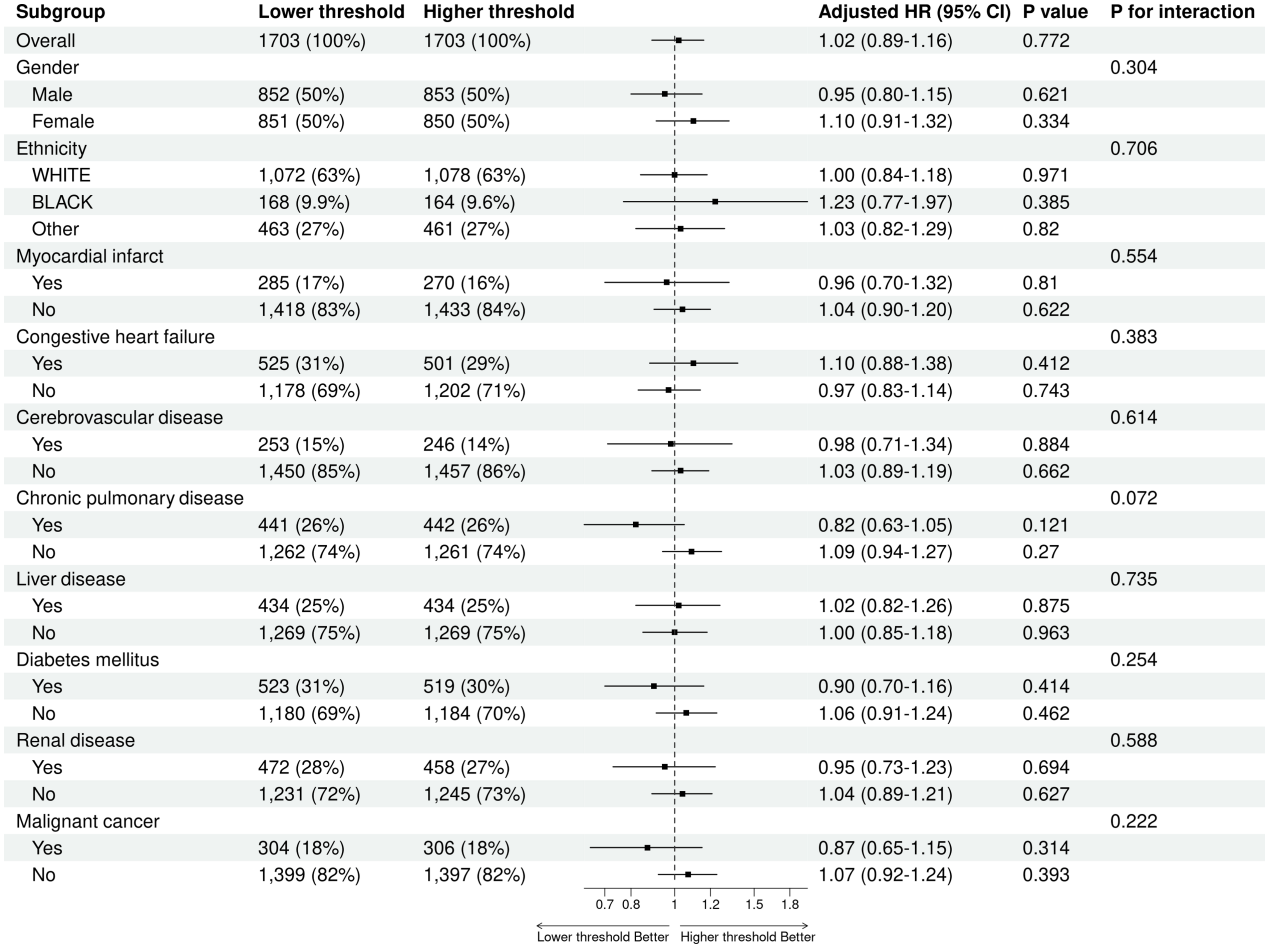
The outcomes of the subgroup analysis.

Figure 6 illustrates the results of the subgroup analysis. Observations within each subgroup, categorized by gender, ethnicity, myocardial infarction, congestive heart failure, cerebrovascular disease, chronic pulmonary disease, liver disease, diabetes mellitus, renal disease, and malignant cancer, led to similar conclusions, indicating no difference in hospital mortality between the two groups.

## Discussion

Our retrospective cohort study investigated the association between various transfusion thresholds and S-AKI prognosis. The analysis showed minimal differences in mortality and ICU stay duration between high and low transfusion threshold groups. Interestingly, despite no significant difference in ICU stays, patients in the lower transfusion threshold group experienced longer hospitalization periods. These findings shed light on the complexity of deciding on transfusion strategies for S-AKI patients, underscoring the need for individualized and context-specific approaches.

Theoretically, sepsis patients exhibit increased basal metabolic rates and oxygen requirements (28). Red blood cell transfusions can potentially correct anemia, enhance renal oxygen delivery, and improve microcirculation, indicating possible benefits (29). However, current research does not entirely corroborate the anticipated outcomes of red blood cell transfusion. Plataki’s et al. (30) study, examining AKI incidence post-transfusion in ICU septic patients, highlighted transfusion’s harmful effects. Repeated transfusions may cause complications such as febrile reactions, acute lung injury, electrolyte imbalances (hyperkalemia and hypocalcemia), and iron overload. Some studies indicate that transfusions, through immune mechanisms and overload, may cause “acute kidney injury” akin to lung injury, potentially leading to or worsening kidney damage (30–32).

Recently, many studies have compared restrictive versus liberal transfusion thresholds (33). The combined data from these studies indicate that, in terms of mortality and adverse reactions, restrictive transfusion thresholds are not inferior to liberal thresholds (34, 35).

Understanding the complex physiological interplay in S-AKI and the subtle effects of different transfusion thresholds may lead to more precise and effective management. Lower transfusion thresholds, not impacting ICU and hospital mortality rates, support the trend towards conservative transfusion in critically ill patients, aligning with guidelines like the “surviving sepsis campaign” (1). However, lower transfusion thresholds could result in prolonged hospital stays, increasing medical costs and complication risks. This may be attributed to the longer recovery time needed for anemic conditions in the low threshold group, or insufficient hemoglobin levels failing to meet clinical needs, causing more complications and extended recovery. Additionally, prolonged hospitalization raises the risk of hospital-acquired infections and other complications. The primary challenge of conservative transfusion strategies (low thresholds) lies in balancing transfusion needs against potential risks. Reducing transfusion frequency may lower complication risks but could delay anemia correction in some patients. This strategy requires finding a balance between reducing unnecessary transfusions and ensuring sufficient blood supply to meet the physiological needs of patients. Future guidelines are expected to provide detailed guidance on transfusion timing and methods, particularly for sepsis with AKI, emphasizing individualized patient assessments to tailor treatment plans.

Naturally, our study has some limitations. Firstly, it is inherently retrospective, which means it may be vulnerable to biases from unmeasured and residual confounding factors, despite our efforts to make comprehensive adjustments for the compared groups. Secondly, the single-center data collection may limit the generalizability of our findings to broader patient populations.

In conclusion, our findings suggest that lower transfusion thresholds may not adversely affect survival in critically ill S-AKI patients, aligning with current clinical thresholds, but could lead to longer hospital stays. Future multicenter, randomized trials are needed to further validate these findings.

Going forward, the paradigm of transfusion will not be merely dichotomized into restrictive or “liberal” strategies. Rather, it will pivot towards a more nuanced approach, tailoring “individualized” transfusion strategies based on the specific disease type, patient characteristics, and prevailing clinical conditions. This shift aims to maximize patients’ long-term prognosis.

## Conclusion

In S-AKI patients, a conservative transfusion strategy with a threshold of <7 g/dL does not adversely affect survival rates compared to a more aggressive approach. However, this approach is linked to longer hospital stays.

## Data availability statement

The raw data supporting the conclusions of this article will be made available by the authors, without undue reservation.

## Ethics statement

The studies involving humans were approved by Boards of the Beth Israel Deaconess Medical Center and Massachusetts Institute of Technology. The studies were conducted in accordance with the local legislation and institutional requirements. Written informed consent for participation was not required from the participants or the participants’ legal guardians/next of kin because written informed consent for participation was not required for this study in accordance with the national legislation and the institutional requirements.

## Author contributions

XR: Writing – original draft, Writing – review & editing. BW: Writing – original draft, Writing – review & editing. YG: Writing – original draft, Writing – review & editing. JW: Writing – original draft, Writing – review & editing. XY: Writing – original draft, Writing – review & editing. CL: Writing – original draft, Writing – review & editing. JP: Writing – original draft, Writing – review & editing.
