# Supplementary material for: Assessing the impact of transfusion thresholds in patients with septic acute kidney injury: a retrospective study
Source: Front Med (Lausanne). 2023 Dec 21;10:1308275. doi: 10.3389/fmed.2023.1308275 (PMC10772139; doi:10.3389/fmed.2023.1308275)
Supplement: Supplementary file 1 [file Data_Sheet_1.docx]

**Assessing the Impact of Transfusion Thresholds in Patients with Septic Acute Kidney Injury: A Retrospective Study**

**SUPPLEMENTAL MATERIAL**

**FigureS1** To reduce the impact of potential confounders, we employed propensity score matching. This statistical technique aimed to balance the covariates between the treatment groups, ensuring that any observed differences in the outcome were more likely to be attributed to the treatments themselves rather than the influence of confounding factors.

The propensity scores were calculated using logistic regression, taking into account the aforementioned demographic and clinical characteristics. We performed 1:1 greedy nearest neighbor matching with a caliper of 0.2. The method functionally relied on the R package MatchIt. A distance was computed between unit of one group and another, and, one by one, each unit was assigned a control unit as a match. The matching was "greedy" in the sense that no action was taken to optimize an overall criterion; each match was selected without considering the other matches that might occur subsequently.

After matching, we performed a comparison of clinical outcomes between the two groups using appropriate statistical tests.


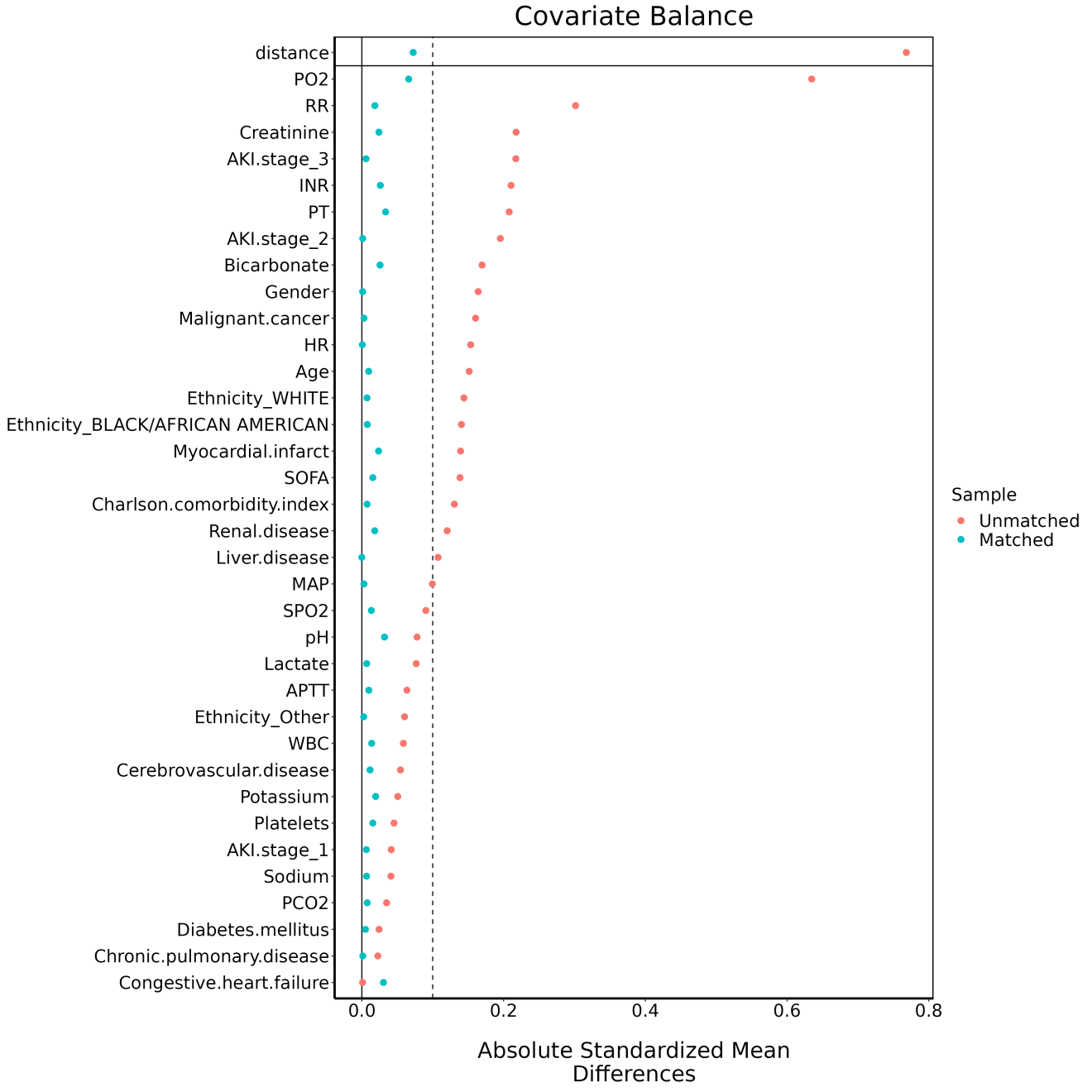


Figure S2. Comparison of the occurrence of Hospital-mortality.


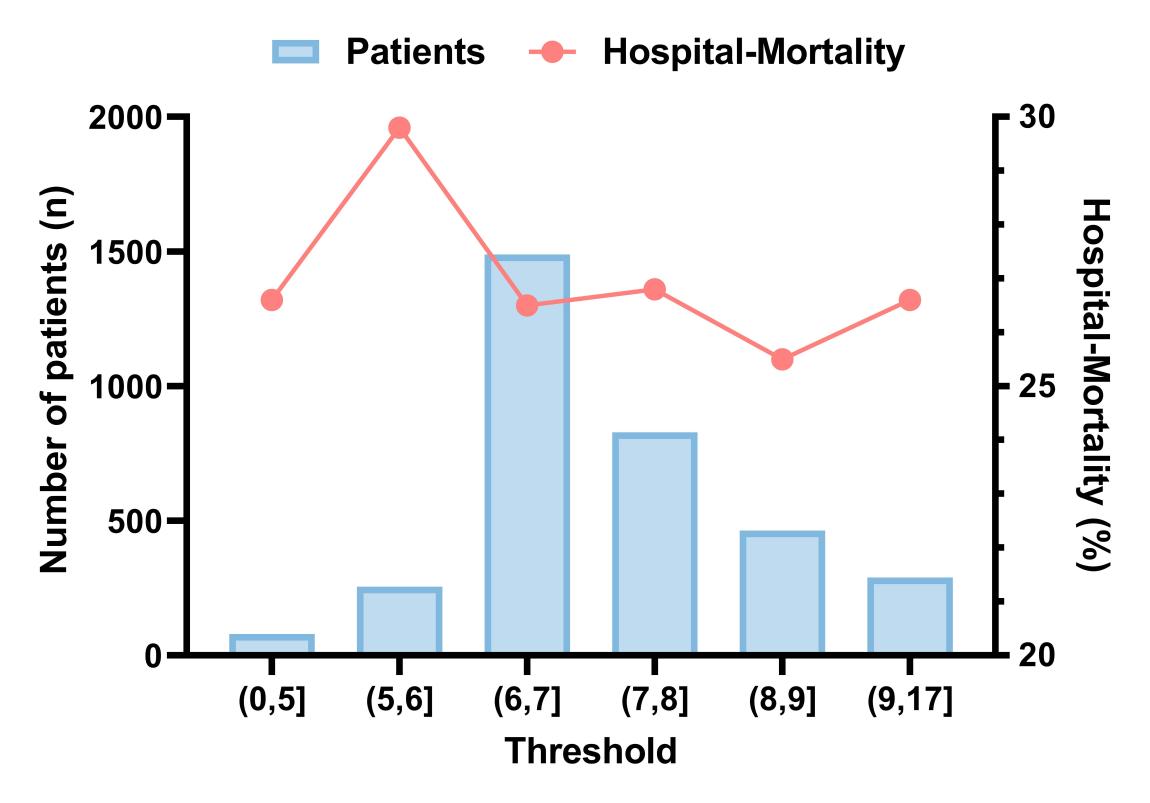


TableS1. Baseline characteristics before matching

| Characteristics | Higher threshold  (N=3873) | Lower threshold  (N=1781) | p |
| --- | --- | --- | --- |
| Age | 67.5 ± 15.2 | 65.0 ± 16.2 | <.001 |
| Male | 2236 (57.7%) | 882 (49.5%) | <.001 |
| Ethnicity |  |  | <.001 |
| WHITE | 2680 (69.2%) | 1108 (62.2%) |  |
| OTHER | 955 (24.7%) | 487 (27.3%) |  |
| BLACK | 238 (6.1%) | 186 (10.4%) |  |
| AKI.stage |  |  | <.001 |
| 1 | 681 (17.6%) | 286 (16.1%) |  |
| 2 | 1725 (44.5%) | 627 (35.2%) |  |
| 3 | 1467 (37.9%) | 868 (48.7%) |  |
| SOFA | 4.2 ± 2.4 | 4.5 ± 2.7 | <.001 |
| HR | 88.7 ± 16.6 | 91.2 ± 16.5 | <.001 |
| RR | 18.9 ± 4.6 | 20.3 ± 4.6 | <.001 |
| MAP | 74.7 ± 11.6 | 73.6 ± 10.7 | <.001 |
| SPO2 | 97.6 ± 2.6 | 97.4 ± 2.7 | .001 |
| Myocardial infarct | 847 (21.9%) | 297 (16.7%) | <.001 |
| Congestive heart failure | 1192 (30.8%) | 549 (30.8%) | .996 |
| Cerebrovascular disease | 3376 (87.2%) | 1518 (85.2%) | .053 |
| Chronic pulmonary disease | 1032 (26.6%) | 457 (25.7%) | .453 |
| Liver disease | 822 (21.2%) | 462 (25.9%) | <.001 |
| Diabetes mellitus | 1144 (29.5%) | 546 (30.7%) | .411 |
| Renal disease | 888 (22.9%) | 505 (28.4%) | <.001 |
| Malignant cancer | 478 (12.3%) | 331 (18.6%) | <.001 |
| WBC | 13.1 ± 8.4 | 14.1 ± 16.6 | .019 |
| Platelets | 179.9 ± 112.0 | 186.3 ± 141.3 | .091 |
| INR | 1.6 ± 0.7 | 1.8 ± 1.0 | <.001 |
| PT | 17.3 ± 7.1 | 19.5 ± 10.9 | <.001 |
| APTT | 43.0 ± 20.2 | 44.4 ± 21.7 | .023 |
| Bicarbonate | 22.7 ± 4.4 | 21.8 ± 5.4 | <.001 |
| Potassium | 4.3 ± 0.6 | 4.3 ± 0.8 | .061 |
| Sodium | 138.3 ± 4.9 | 138.5 ± 6.1 | .128 |
| Creatinine | 1.6 ± 1.5 | 2.0 ± 2.0 | <.001 |
| pH | 7.4 ± 0.1 | 7.4 ± 0.1 | .005 |
| PO2 | 173.8 ± 87.1 | 125.6 ± 76.0 | <.001 |
| PCO2 | 40.5 ± 7.0 | 40.2 ± 8.5 | .197 |
| Lactate | 2.7 ± 1.9 | 2.8 ± 2.5 | .004 |

Table S2. Baseline characteristics after matching

| Characteristics | Higher threshold  (N=1703) | Lower threshold  (N=1703) | p |
| --- | --- | --- | --- |
| Age | 65.2 ± 15.9 | 65.1 ± 16.3 | .775 |
| Male | 853 (50.1%) | 852 (50%) | 1.000 |
| Ethnicity |  |  | .966 |
| WHITE | 1078 (63.3%) | 1072 (62.9%) |  |
| OTHER | 461 (27.1%) | 463 (27.2%) |  |
| BLACK | 164 (9.6%) | 168 (9.9%) |  |
| AKI.stage |  |  | .978 |
| 1 | 270 (15.9%) | 274 (16.1%) |  |
| 2 | 609 (35.8%) | 610 (35.8%) |  |
| 3 | 824 (48.4%) | 819 (48.1%) |  |
| SOFA | 4.5 ± 2.6 | 4.5 ± 2.6 | .647 |
| HR | 91.3 ± 17.7 | 91.2 ± 16.5 | .984 |
| RR | 20.2 ± 4.9 | 20.3 ± 4.6 | .600 |
| MAP | 73.9 ± 11.0 | 73.9 ± 10.6 | .931 |
| SPO2 | 97.4 ± 2.2 | 97.3 ± 2.7 | .672 |
| Myocardial infarct | 270 (15.9%) | 285 (16.7%) | .516 |
| Congestive heart failure | 501 (29.4%) | 525 (30.8%) | .390 |
| Cerebrovascular.disease | 246 (14.4%) | 253 (14.9%) | .771 |
| Chronic.pulmonary.disease | 442 (26%) | 441 (25.9%) | 1.000 |
| Liver disease | 434 (25.5%) | 434 (25.5%) | 1.000 |
| Diabetes mellitus | 519 (30.5%) | 523 (30.7%) | .911 |
| Renal disease | 458 (26.9%) | 472 (27.7%) | .617 |
| Malignant.cancer | 306 (18%) | 304 (17.9%) | .964 |
| WBC | 13.8 ± 10.4 | 14.1 ± 16.7 | .628 |
| Platelets | 188.8 ± 131.1 | 186.6 ± 140.4 | .639 |
| INR | 1.7 ± 0.9 | 1.8 ± 0.9 | .398 |
| PT | 18.7 ± 9.1 | 19.0 ± 9.8 | .261 |
| APTT | 44.1 ± 21.8 | 43.9 ± 21.0 | .770 |
| Bicarbonate | 22.0 ± 4.8 | 21.9 ± 5.4 | .425 |
| Potassium | 4.3 ± 0.7 | 4.3 ± 0.8 | .546 |
| Sodium | 138.5 ± 5.4 | 138.4 ± 6.0 | .835 |
| Creatinine | 1.9 ± 1.8 | 2.0 ± 2.0 | .450 |
| pH | 7.4 ± 0.1 | 7.4 ± 0.1 | .339 |
| PO2 | 132.9 ± 70.2 | 127.9 ± 76.5 | .046 |
| PCO2 | 40.2 ± 8.1 | 40.2 ± 8.5 | .820 |
| Lactate | 2.8 ± 2.3 | 2.8 ± 2.5 | .832 |

Table S3 :Univariate and multivariate analysis of length of stay.

|  | **Univariable** | | | | **Multivariable** | | | |
| --- | --- | --- | --- | --- | --- | --- | --- | --- |
| **Characteristic** | **N** | **Beta** | **95% CI**^1^ | **p-value** | **N** | **Beta** | **95% CI**^1^ | **p-value** |
| Gender |  |  |  |  |  |  |  |  |
| Male | 1,705 | — | — |  | 1,705 | — | — |  |
| Female | 1,701 | -0.12 | -0.63, 0.38 | 0.626 | 1,701 | -0.15 | -0.66, 0.36 | 0.571 |
| Ethnicity |  |  |  |  |  |  |  |  |
| WHITE | 2,150 | — | — |  | 2,150 | — | — |  |
| BLACK | 332 | 0.55 | -0.32, 1.41 | 0.216 | 332 | 0.49 | -0.39, 1.36 | 0.275 |
| Other | 924 | 0.10 | -0.48, 0.68 | 0.738 | 924 | 0.09 | -0.49, 0.67 | 0.770 |
| Group |  |  |  |  |  |  |  |  |
| Lower threshold | 1,703 | — | — |  | 1,703 | — | — |  |
| Higher threshold | 1,703 | -0.58 | -1.08, -0.08 | 0.023 | 1,703 | -0.58 | -1.08, -0.07 | 0.024 |
| Myocardial infarct |  |  |  |  |  |  |  |  |
| Yes | 555 | — | — |  | 555 | — | — |  |
| No | 2,851 | 0.69 | 0.01, 1.37 | 0.047 | 2,851 | 0.81 | 0.09, 1.53 | 0.027 |
| Congestive heart failure |  |  |  |  |  |  |  |  |
| Yes | 1,026 | — | — |  | 1,026 | — | — |  |
| No | 2,380 | -0.29 | -0.84, 0.26 | 0.298 | 2,380 | -0.51 | -1.11, 0.09 | 0.095 |
| Cerebrovascular disease |  |  |  |  |  |  |  |  |
| Yes | 499 | — | — |  | 499 | — | — |  |
| No | 2,907 | -1.28 | -1.99, -0.57 | <0.001 | 2,907 | -1.35 | -2.07, -0.64 | <0.001 |
| Chronic pulmonary disease |  |  |  |  |  |  |  |  |
| Yes | 883 | — | — |  | 883 | — | — |  |
| No | 2,523 | -0.18 | -0.75, 0.40 | 0.549 | 2,523 | -0.18 | -0.76, 0.40 | 0.548 |
| Liver disease |  |  |  |  |  |  |  |  |
| Yes | 868 | — | — |  | 868 | — | — |  |
| No | 2,538 | -0.47 | -1.05, 0.10 | 0.107 | 2,538 | -0.56 | -1.15, 0.03 | 0.061 |
| Diabetes mellitus |  |  |  |  |  |  |  |  |
| Yes | 1,042 | — | — |  | 1,042 | — | — |  |
| No | 2,364 | 0.20 | -0.34, 0.75 | 0.469 | 2,364 | 0.20 | -0.38, 0.78 | 0.506 |
| Renal disease |  |  |  |  |  |  |  |  |
| Yes | 930 | — | — |  | 930 | — | — |  |
| No | 2,476 | 0.04 | -0.52, 0.61 | 0.886 | 2,476 | 0.07 | -0.54, 0.68 | 0.832 |
| Malignant cancer |  |  |  |  |  |  |  |  |
| Yes | 610 | — | — |  | 610 | — | — |  |
| No | 2,796 | -0.22 | -0.88, 0.43 | 0.506 | 2,796 | -0.28 | -0.95, 0.38 | 0.399 |
| ^1^CI = Confidence Interval | | | | | | | | |
| Number in dataframe = 3406, Number in model = 3406, Log-likelihood = -11672.5, AIC = 23373, R-squared = 0.0091, Adjusted R-squared = 0.0056 | | | | | | | | |

**The diagnosis of AKI**

AKI: AKI followed the KDIGO definition: urine output less than 0.5 ml/kg/hour for 6 hours, or serum creatinine (SCr) increases >= 0.3 mg/dl within 48 hours or increases >= 1.5 times baseline value within 7 days. The stage of AKI was also in according with KDIGO criteria.
